# Evaluation of the impact of immunization policies, including the addition of pharmacists as immunizers, on influenza vaccination coverage in Nova Scotia, Canada: 2006 to 2016

**DOI:** 10.1186/s12889-018-5697-x

**Published:** 2018-06-26

**Authors:** Jennifer E. Isenor, Beth A. O’Reilly, Susan K. Bowles

**Affiliations:** 1 College of Pharmacy, Faculty of Health, Dalhousie University, 5968 College Street, PO Box 15000, Halifax, NS B3H 4R2 Canada; 2 Canadian Center for Vaccinology, Nova Scotia Health Authority, IWK Health Centre, Dalhousie University, Halifax, NS Canada; 3 Department of Pharmacy, Nova Scotia Health Authority, 1276 South Park Street, Halifax, NS B3H 2Y9 Canada

**Keywords:** Influenza, Vaccination, Public health, Policy, Physician immunizers, Pharmacist immunizers, Canada

## Abstract

**Background:**

Influenza is a serious public health concern, resulting in morbidity, mortality and significant expense to healthcare systems worldwide. Annual vaccination is the most effective way to prevent influenza. The National Advisory Committee on Immunization in Canada recommends that everyone six months of age and older without contraindications should be vaccinated. The Canadian province of Nova Scotia implemented a publicly-funded universal influenza vaccination program in the 2010–2011 influenza season. In 2013, pharmacists in Nova Scotia gained the authority to provide a variety of vaccinations, including the publicly-funded influenza vaccine. This study aimed to investigate any changes in influenza vaccine coverage following the implementation of each policy change: 1) universal publicly-funded program and 2) universal publicly-funded program with the addition of pharmacists.

**Methods:**

Influenza seasons evaluated were from 2006-2007 to 2015–2016. Coverage was estimated by examining Nova Scotia census data with aggregate immunization administration data, including the total number of vaccinations administered according to vaccine provider (physician, public health or pharmacist), geographic region, vaccine recipient age and year.

**Results:**

The analysis showed an increase in influenza vaccine coverage immediately following the implementation of the two studied policy changes. Vaccine coverage increased from 36.4 to 38% following the implementation of the universally funded vaccine policy. Following the implementation of pharmacists as immunizers, coverage increased from 35.7 to 41.7%. Vaccine coverage was highest in those 65 years of age and older during all years evaluated. Physicians provided the highest proportion of vaccines during all study periods, however a decreasing trend through all periods was observed. Physicians proportionately provided more vaccines in urban areas; whereas pharmacist and public health immunization providers in rural areas provided proportionately more vaccinations than their urban counterparts.

**Conclusions:**

The addition of a universally funded vaccination policy and the addition of pharmacists as providers of the influenza vaccine resulted in increases in vaccine coverage initially. Additional research is needed to determine the long-term impacts of the policy changes on vaccination coverage and to identify other important factors affecting vaccine uptake.

## Background

Influenza is a serious public health concern, associated with severe illness and death, particularly in high-risk populations [1]. Worldwide, an estimated 3 to 5 million serious cases and 250,000 to 500,000 deaths are attributed on average to influenza annually [1]. Within Canada, it is estimated that 10 to 20% of the population is infected with influenza each year, resulting in an average of 12,000 hospitalizations and 3500 deaths annually [2, 3].

Annual influenza vaccine is the most effective method to prevent influenza and its complications [1, 3]. The Canadian National Consensus Conference for Vaccine Preventable Diseases in Canada proposed targets for influenza vaccine coverage, ranging from 80 to 95%, for high-risk individuals and healthcare professionals, respectively [3, 4]. Furthermore, as significant illness and high societal costs can occur in those even without high-risk complications, the National Advisory Committee on Immunization (NACI) recommends the influenza vaccine for all Canadians six months of age and older [3].

A variety of influenza immunization strategies have been utilized, with many jurisdictions focusing on vaccinating high-risk populations, such as older individuals, children and those with chronic disease, and others providing universal coverage [5–8]. Although NACI in Canada recommends the vaccine for all Canadians six months of age and older [3], public funding for the influenza vaccine varies by province/territory, as each is responsible for providing healthcare to their residents [9, 10]. The addition of non-traditional providers, such as pharmacists, in non-clinic settings has also been considered as a method to improve coverage through improved convenience and possibly access with more providers, more convenient locations, and extended service availability (evenings and weekends) [11–13].

The Nova Scotia Department of Health and Wellness (DHW) initiated a universal influenza program for residents in an effort to improve vaccination uptake in the 2010–2011 influenza season. [14, 15]. This was followed by legislation implemented during the 2013–2014 influenza season enabling pharmacists who have taken appropriate training (theory modules, exam and practical assessment) and obtained a permit to provide publicly funded influenza vaccine to patients five years of age and older [16]. Pharmacies receive a modest fee to cover the administration costs (supplies and pharmacist time) [17]. The goal of these changes was to improve vaccine uptake by eliminating cost as a barrier and increasing convenience and possibly access; however, the impact of these changes has not been fully explored.

This study aims to compare influenza vaccine coverage between three different policy periods: 1) pre-universal influenza vaccination program; 2) universal publicly funded program; and 3) universal publicly funded program with the addition of pharmacists.

## Methods

Census data and aggregate immunization data were obtained from the Nova Scotia DHW. This data included the total number of vaccinations administered according to vaccine provider (physician, public health, or pharmacist), geographic region according to health authority zone, recipient age, and year (end of August to the end of August the following year).

For the purpose of comparison, 2006–2007, 2007–2008 and 2008–2009 were defined as the pre-universally funded program seasons; 2010–2011, 2011–2012 and 2012–2013 were defined as universally funded program, pre-pharmacist participation seasons; 2013–2014, 2014–2015 and 2015–2016 were universally funded program with the addition of pharmacist seasons. Due to pandemic H1N1 in 2009–2010, Public Health did not collect seasonal influenza vaccination data, so this season was omitted from comparisons and described separately.

Aggregate provider data was not available for the 2008–2009 season. The vaccine coverage of the overall community-dwelling population aged 6 months of age and older for this season was estimated using the Public Health distribution of influenza vaccine to all providers subtracted by the number of vaccines that were returned at the end of the vaccinating season.

Estimated vaccination coverage rates for influenza vaccine were constructed for each year, using the number of influenza vaccinations administered as the numerator and the census data closest to the year of interest as the denominator. Vaccination coverage was calculated using total populations and estimates grouped by age and location. Coverage rates were compared between vaccination provider groups. Analysis based on geographic location (urban versus rural) was based on Nova Scotia Health Authority (NSHA) management zones. The comparisons between the proportions of the immunized populations were made using Chi-Square with Bonferroni correction for multiple comparisons. All data analysis was completed using EXCEL version 14.4.7.

## Results

**Fig. 1:**
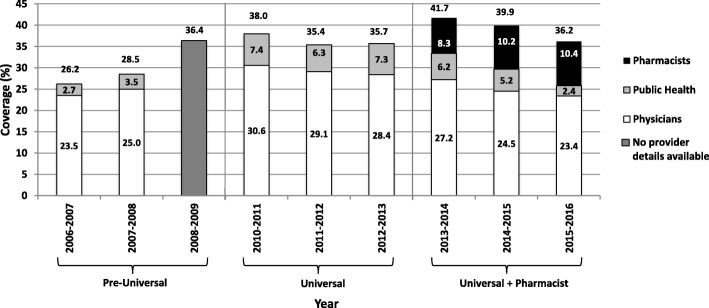
Influenza vaccination coverage for Nova Scotia residents aged six months of age and older (2006–2016)

Vaccine coverage by year and provider for community-dwelling residents six months of age and older are shown in Fig. 1. Following the addition of the universal funding policy, coverage increased to 38% from a previous high of 36.4%. The 2013–2014 season was the first that pharmacists were permitted to vaccinate, which saw vaccination coverage rise again to 41.7%, its highest point in the study periods.

**Fig. 2:**
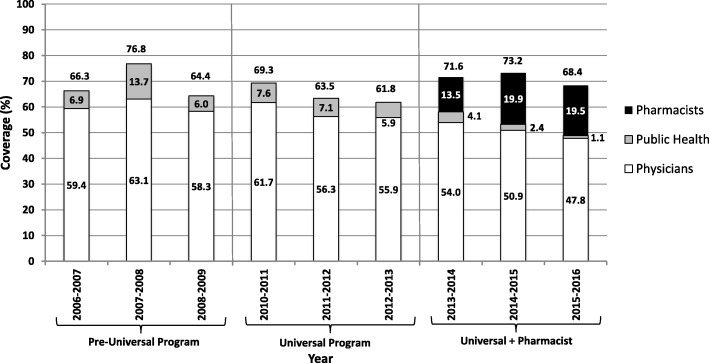
Influenza vaccination coverage for Nova Scotia community-dwelling residents aged 65 years of age and older (2006–2016)

**Fig. 3:**
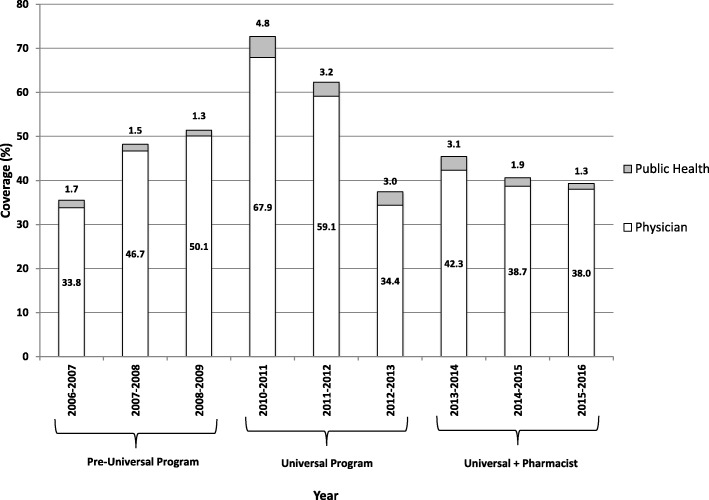
Influenza vaccination coverage for Nova Scotia infants* *Aged 6 to 24 months for 2006–2007 to 2011–2012 and 6 to 59 months for 2012–2013 to 2015–2016.

Influenza vaccine coverage by age groups are shown in Figs. 2 and 3. Coverage for community-dwelling adults aged 65 years of age and older ranged from a high of 76.8% in 2007–2008 and a low of 61.8% in 2012–2013. Data for Nova Scotia children changed from being grouped as 6 to 24 months to children 6 to 59 months in the 2012–2013 vaccinating season, making direct comparisons for this population challenging; however, a steady increase in coverage was seen from 2006-2007 to 2010–2011, with a decline noted in the years following (Fig. 3).

**Fig. 4:**
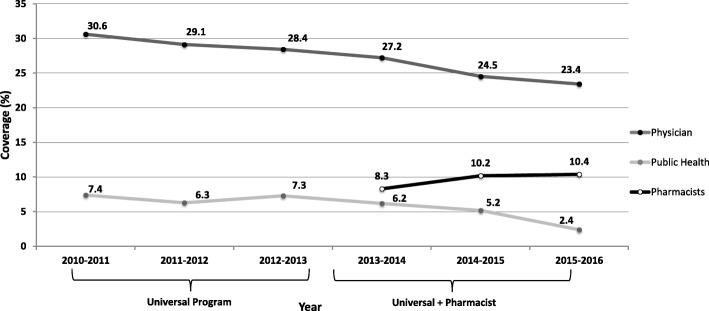
Trends in influenza vaccination coverage in Nova Scotia by provider (2010–2016)

Influenza vaccine coverage by physicians has been steadily declining since the implementation of the universally funded policy, whereas coverage provided by pharmacists has increased since they were permitted to vaccinate (Fig. 4). Administration by public health remained relatively consistent until a steady decline was observed after the 2012–2013 season (Fig. 4).

**Fig. 5:**
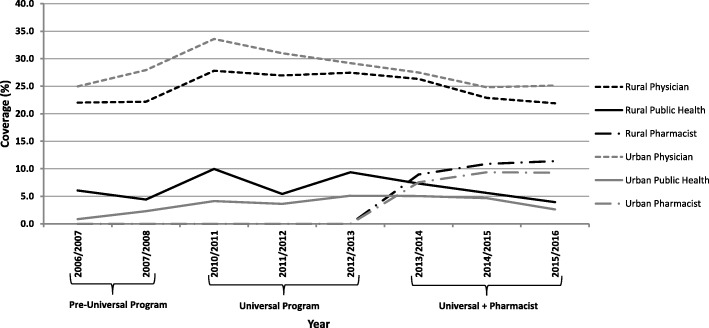
Trends in influenza vaccination coverage by geographic location and provider in Nova Scotia (2006–2016)

Trends in vaccine coverage according to rural versus urban status showed that proportionately more physicians in urban settings are providing influenza vaccinations compared to those in rural settings (Fig. 5). This differed from pharmacist and public health providers, which were both found to provide proportionately more vaccines in rural settings compared to urban settings (Fig. 5).

Minimal data was collected during the 2009 H1N1 pandemic in Nova Scotia, however, it was confirmed that at least 54% of Nova Scotia residents were vaccinated against H1N1 [18]. Vaccine coverage varied by age group, with the highest coverage in those 6 to 35 months of age, and the lowest coverage observed in those 20 to 24 years of age. An estimated 64% of pregnant women in the province of Nova Scotia were immunized against H1N1 [18].

## Discussion

Our analysis demonstrated an increase in influenza vaccine coverage immediately following the implementation of the two policy changes.

The addition of a universal immunization policy in 2010–2011 led to an increase in vaccination coverage from 36.4 to 38%. This result is smaller than that observed in Ontario, another Canadian jurisdiction that offers universal influenza immunization [19]. They observed an increase in the immunization rate from 18% in 1996–1997 to 35% in 2003–2004, while the other provinces combined experienced an increase from 13 to 23% [19]. The greatest increase was seen in those between 12 and 65 years of age, which we did not observe in our data [19].

Despite the initial increase in coverage observed, a reduction in coverage was observed in the two years following. The higher vaccine coverage in the 2010–2011 season may have been due to increased awareness and access to the vaccine, since this was the first year the vaccine was publicly funded universally. However, the increase could also be attributed to the residual impact of the H1N1 pandemic the year before, which was also noted in one European study [20]. The 2013–2014 season was the highest vaccination coverage seen during the study seasons, at 41.8%, which was also the first year that pharmacists provided influenza immunization services in Nova Scotia. Despite the initial increase following the addition of pharmacists as immunizers, influenza vaccine coverage steadily declined in the two years following implementation of the pharmacist policy, with the reasons for the decline unclear. This differs from results in a study from the United States, where the odds that an adult received the influenza vaccine rose with the addition of pharmacists as immunizers and this effect continued to increase over time [21]. One possible explanation why our data seems to differ is the vaccine mismatch observed in the 2014–2015 season, leading to public distrust in the vaccine’s efficacy resulting in a decreased uptake in the 2015–2016 season [22]. A German study found that some older adults did not receive the influenza vaccine because they didn’t trust it [23]. However, as the population becomes more confident in influenza vaccine efficacy, an overall increase in influenza immunization rates may be seen in subsequent years. A US study found that higher perceived benefits were an independent predictor for vaccine uptake [24]. It is also possible that by increasing access through pharmacies, overall vaccination rates may not have fallen as far as they might have had pharmacists not been able to provide this service.

Coverage in individuals aged 65 years of age and older remained relatively consistent with the addition of a universally funded vaccination program compared to the pre-universal study years. The influenza vaccine was publicly funded for this age group prior to the policy change, therefore, the change was not expected to significantly affect this age group. This population are also more likely to visit their physician on a regular basis, so may be offered vaccination more often. The highest coverage for this patient population was observed in the 2007–2008 and the reason for the high coverage in this year is not clear, despite conversations with DHW staff and review of media reports from that time, and may therefore be related to inaccurate data reporting. Influenza activity in the previous season (2006–2007) was relatively low worldwide, further supporting that media coverage was not likely to account for the high coverage [25]. The addition of pharmacists led to increased coverage during the first two years, followed by a decrease in the third year; however, the coverage in this age group by pharmacists stayed high in the third year with only a slight decrease to 19.5% from 19.9% the previous year. Physicians and Public Health saw larger declines in provision of vaccines in this age group. The decline in provision by physicians may be part of the ongoing overall decline in provision of influenza vaccines by physicians, which was seen throughout the study years. The reasons for this decrease are not fully understood and likely multifactorial. Many physicians express concern about inadequate time to spend with each patient, which may result in focusing on the acute issue and less time to consider preventative strategies, such as vaccinations [26]. Additionally, there is a perceived shortage of family physicians in Nova Scotia being identified as a barrier to patients receiving care, which could directly lead to fewer vaccinations being administered by physicians [27]. The decline in coverage by Public Health was planned in anticipation of the addition of pharmacists as immunizers so that Public Health resources could be utilized for other health care measures.

Influenza vaccine coverage in infants was found to have been steadily increasing throughout the pre-universal program period, with a large increase with the addition of the universal policy in 2010–2011 after which coverage declined for the remainder of the study period. This is consistent with data from Ontario, Canada which observed low influenza coverage rates in children aged 6 to 23 months, despite implementation of a universal vaccination program and a high rate of primary care visits [28]. However, one important consideration in our study is that aggregate data from 2006-2007 to 2011–2012 was for ages 6 to 24 months, whereas the 2012–2013 to 2015–2016 seasons were for ages 6 to 59 months. Another consideration is that in Nova Scotia, pharmacists are only permitted to vaccinate individuals over the age of 5; therefore, they are not involved with the vaccination of infants and young children against influenza. As seen in Fig. 3, physicians primarily immunize this age group, so the possible reasons for decreased physician provision of vaccinations previously identified above may be contributing to a decline in this age group as well.

The impact of the policy changes on vaccine coverage by geographic location indicated similar trends to overall coverage. However, of note, we observed proportionately more physicians in urban areas providing influenza vaccines than rural physicians and conversely proportionately more rural pharmacists and public health providers administering influenza vaccines compared to their urban counterparts. In the province of Nova Scotia, nearly 40,000 residents are without a primary care provider, with over 19,000 of those residents living in rural communities [29]. Rural communities in Nova Scotia tend to experience substantial deficits in the provision of health services, which may explain the decreased provision of influenza vaccinations by physicians in these areas, and may continue as the number of Nova Scotia residents without a primary care provider increases [30]. A retrospective study from the United States found pharmacists provided the influenza vaccine to the majority (up to 71%) of residents in medically underserved areas, which supports our results in finding pharmacists providing proportionately more vaccines in rural areas [31]. The results are further supported by a small cluster-randomized trial in rural areas in the province of British Columbia, which demonstrated that pharmacy-based influenza vaccination clinics resulted in increased vaccination rates for residents older than 65 years of age [32].

Research regarding the impact that pharmacists as immunizers have on vaccine coverage is growing. A recent systematic review and meta-analysis found that pharmacist administration of vaccines resulted in increased uptake of immunizations [33]. In the United States, studies have shown that adults aged 65 years of age and older had higher influenza vaccination uptake in states where pharmacists were permitted to vaccinate compared to those that did not have pharmacists as immunizers [21, 34]. Additionally, one study found increased influenza vaccine coverage in young adults with pharmacists providing immunizations and another observed improved coverage in rural populations in the United States with pharmacists immunizing [35, 36]. Research from England found pharmacist provision of immunizations improved vaccination rates; was considered convenient and accessible and in one study many patients chose to pay for the service in the pharmacy despite being eligible for free vaccine [37–39]. Pharmacist services were used by patients from all socio-demographic areas, and especially caregivers, healthcare workers and those of working age [38]. Previous research from Nova Scotia suggested that the addition of pharmacists as immunizers within the publicly funded influenza program increased overall vaccine coverage in the first two years that included pharmacists as immunizers in addition to universal funding [40, 41]. This study was able to look at additional data, including data prior to universal funding, geographic distribution data, as well as an additional year of pharmacists as immunizers. It was unclear if pharmacists were capturing new, previously unimmunized patients or if it was a shift in provider in the earlier studies. The ongoing trend of declines in provision of influenza vaccines by physicians is clearer with this study. Additional research will be required to determine whether it is related to a decrease in primary care providers or if there are other factors and the potential role pharmacists can play in addressing these concerns.

Limitations for this study include potential errors related to the use of secondary aggregate data from health claims, including age-related coding, geographic location coding, or improper billing. Although census data was not perfectly matched between each influenza season, the growth and changes between years were small and consistent and are not expected to impact the results. Missing data from the 2008–2009 season prevented complete comparison of the overall population according to provider type. When assessing vaccinations for patients less than 9 years of age, it is not possible to determine the proportion of those patients that received the recommended two doses of the vaccine if it was their first year being vaccinated. However, over-reporting of the double dose would be consistent in all years and all providers, so the overall impact on the results would be expected to be minimal. Short time frames of three-years for each period may have prevented evaluating the full impact of each policy long-term; however, as the universal policy was only in place for three years, prior to the addition of pharmacists, a longer study was not possible. Additional potential confounders may include the 2009 H1N1 pandemic leading to increased coverage in the 2010–2011 season, a perceived vaccine shortage in 2013–2014 with unknown effects on coverage, and decreased public confidence in the vaccine for the 2015–2016 season due to a mismatched vaccine in the previous year potentially led to the decreased coverage seen. There is the potential for privately purchased vaccines that were not funded within the public program (e.g. adjuvanted) to affect the results; however, this is unlikely as there were no preferential recommendations [3].

Our results support previous research that vaccine uptake is a multifactorial process, and that factors other than cost and convenience are involved in vaccine uptake by the public [42, 43]. The new policy changes during the study periods resulted in an overall increase in vaccine coverage, however the exact reasons for that are difficult to determine. Possible suggestions include the lack of cost of the vaccine, increased convenience, increased advertising during the time of the new policy periods, or by patients being initially enthusiastic about the new policies leading to increased uptake. Despite the overall increase, vaccine coverage was not sustained during the years following, which again, could be multifactorial. One possible reason for the decline in coverage could be complacency by the public, particularly if the previous influenza season did not receive a lot of media attention, or if it did not result in high morbidity or mortality. Additionally, further research into what other barriers exist that prevent individuals from seeking their annual influenza vaccine is required. This study aimed to look at the impact of two previously identified barriers to vaccinations, convenience and cost, therefore further research is required to clearly identify other perceived barriers and the impact of strategies to improve them on immunization coverage.

## Conclusions

The additions of a universal influenza vaccination policy and pharmacists as immunizers within the influenza vaccination program in Nova Scotia, Canada led to increased influenza vaccination coverage initially. Possible explanations include no out-of-pocket cost for the vaccine, increased media coverage for the publicly funded vaccine and the addition of pharmacists, and improved convenience with additional providers, locations, and extended service hours, or a combination of these factors. Additional research is needed to determine the long-term impacts of the policy changes on vaccination coverage, as well as evaluation of other important factors affecting vaccine uptake.

## Abbreviations

DHA: District Health Authorities
DHW: Department of Health and Wellness
NACI: National Advisory Committee on Immunization
NSHA: Nova Scotia Health Authority
